# The 46.1 Antibody Mediates Neurotensin Uptake into the CNS and the Effects Depend on the Route of Intravenous Administration

**DOI:** 10.3390/pharmaceutics14081706

**Published:** 2022-08-16

**Authors:** Julia V. Georgieva, Moriah Katt, Zhou Ye, Benjamin J. Umlauf, Cody J. Wenthur, Eric V. Shusta

**Affiliations:** 1 Department of Chemical and Biological Engineering, University of Wisconsin-Madison, Madison, WI 53706, USA; 2 Divisions of Pharmaceutical Sciences and Pharmacy Practice, School of Pharmacy, University of Wisconsin-Madison, Madison, WI 53705, USA; 3 Department of Neurological Surgery, University of Wisconsin-Madison, Madison, WI 53792, USA

**Keywords:** blood-brain barrier, brain drug delivery, receptor-mediated transcytosis, antibody, cavernous sinus

## Abstract

Central nervous system (CNS) exposure to blood-borne biotherapeutics is limited by the restrictive nature of the brain vasculature. In particular, tightly sealed endothelial cells of the blood–brain barrier (BBB) prevent the uptake of protein and gene medicines. An approach to increase the bioavailability of such therapeutics is harnessing the BBB endothelial cells’ own receptor-mediated transcytosis (RMT) mechanisms. Key to this process is a targeting ligand that can engage a BBB-resident RMT receptor. We recently identified an antibody, named 46.1, that accumulates in the mouse brain after intravenous injection. To further characterize the brain targeting and penetrating properties of clone 46.1, we conjugated neurotensin (NT) to an scFv-Fc form of the antibody (46.1-scFv-Fc-**L**ong**L**inker-NT). While centrally administered NT decreases the core body temperature and locomotor activity, effects attributed to two spatially segregated brain areas, systemically administered NT has limited effects. Hence, NT can be used as a model therapeutic payload to evaluate the brain penetration of BBB-targeting antibodies and their capability to accumulate in discrete brain areas. We demonstrate that intravenously administered 46.1-scFv-Fc-LL-NT can elicit transient hypothermia and reduce drug-induced hyperlocomotion, confirming that 46.1 can deliver drug cargo to the CNS at pharmacologically relevant doses. Interestingly, when two intravenous administration routes in mice, retro-orbital and tail vein, were compared, only retro-orbital administration led to transient hypothermia. We further explored the retro-orbital route and demonstrated that the 46.1-scFv-Fc-LL-NT could enter the brain arterial blood supply directly from the retro-orbital/cavernous sinus. Taken together, the 46.1 antibody is capable of transporting drug cargo into the CNS, and at least of a portion of its CNS accumulation occurs via the cavernous sinus–arterial route.

## 1. Introduction

Drug delivery into the brain remains the rate limiting step in the development of new therapies whose targets lie within the central nervous system (CNS). In particular, the passage of newer biologic therapeutics (antibodies, peptides, nucleic acids, etc.) from the systemic circulation into the brain is substantially restricted by the blood–brain barrier (BBB) [1]. As a result, various technologies are being developed to increase the brain bioavailability. Despite known limitations [2], those that co-opt endogenous receptor-mediated transcytosis (RMT) systems in BBB endothelial cells hold particular promise [3,4,5,6,7,8]. The transport of a drug payload from the blood into the brain tissue by RMT is mediated by a BBB-targeting motif that recognizes a cognate receptor on the blood-side endothelial membrane and initiates transcytosis. In preceding work, we identified antibodies capable of BBB transcytosis using a phenotypic transcytosis screen of a large phage displayed human single-chain antibody (scFv) library [9]. The lead molecule, scFv 46.1, bound mouse and human BBB in tissue sections and accumulated in the mouse brain parenchyma after intravenous administration.

A key step in the preclinical evaluation of BBB-targeting motifs is demonstrable transport of a drug payload into the brain. Advanced disease models in translational research such as those examining β-amyloid clearance have been used to demonstrate the uptake of pharmacologically relevant doses of the therapeutic using RMT-directed brain drug delivery approaches [7]. Other strategies that are more focused on validating the RMT-targeted antibody as capable of mediating drug uptake into the CNS, rather than a therapeutic outcome, have also been used to validate RMT-targeting antibodies [10]. One such approach employs the conjugation of the RMT-targeting antibody to neurotensin (NT), a 13 amino acid peptide with a myriad of physiological functions. If administered peripherally, NT has a very low BBB permeability [11] and does not elicit profound effects in the CNS. However, if NT is introduced into the CNS, it can interact with NT receptors expressed on brain cells (NTSR1 and NTSR2) in different brain regions. For example, NT and its analogs inhibit food intake in arcuate nucleus [12], modulate pain response [13], and mitigate addiction behavior in nucleus accumbens [14,15,16]. In addition, NT integrates with dopamine neurotransmission, acting as an endogenous neuroleptic, leading to decreased drug-induced hyper- and spontaneous locomotor activity [17,18]. Upon release in the median preoptic nucleus (MnPO), NT activates its cognate receptors NTSR1 and NTSR2, which results in decreased core body temperature [19]. Since the CNS effects of NT are limited to central local release or central administration, fusion of NT to BBB-targeting antibodies can be used to test the BBB-permeation of the complex [8,10,11]. Notably, given the anatomically distinct effects of NT, it can also be used as a proxy for verifying brain uptake in different brain regions. For instance, in this study, we measured the effects of 46.1-NT fusions on the body temperature in mice (i.e., NT response from the MnPO), and additionally measured their drug-induced locomotor activity (i.e., response from the striatum) to demonstrate that 46.1 could mediate the uptake of pharmacologically relevant levels of NT in the CNS. 

It has been shown that the pharmacokinetic profile of intravenous therapeutic antibodies can be independent of the route of intravenous administration [20]. Nevertheless, viral particles with CNS tropism seem to distribute differently upon tail vein, facial vein, or retro-orbital vein injection [21,22,23], a phenomenon speculated to arise in the cavernous sinus (Sin. Cavern.). The cavernous sinus consists of trabeculated cavities formed by splitting of the layers of the dura mater that are covered by endothelial cells. Located in the base of the skull, right below the hypophyseal gland, the cavernous sinus collects venous blood from the retro-orbital sinus, ophthalmic veins, superficial, and interior cerebral veins. The internal carotid artery spans the Sin. Cavern. before branching and entering the brain in a segment known as the cavernous portion of the internal carotid artery [24]. Thus, in the Sin. Cavern., venous blood is separated from the arterial blood by the wall of the internal carotid artery, a multilayered structure with an innermost endothelial cell layer, covered by internal elastic lamina, smooth muscle cell layer, and adventia [25,26]. In this study, we additionally examined the effects of the route of intravenous administration (retro-orbital versus tail vein) on the hypothermic response in mice and demonstrated that the 46.1-NT fusion could enter the internal carotid artery directly from the retro-orbital/cavernous sinus and be transported throughout the brain via the microvasculature.

## 2. Materials and Methods

### 2.1. Construction of Neurotensin–Antibody Fusion Proteins

A previously described pIRES vector with the test and control scFvs fused to the rabbit Fc region was used to generate the neurotensin fusion construct [9]. Long linker (G_4_S)_2_ and mouse neurotensin sequences (NM_024435) were subcloned at the C-terminal of the rabbit Fc region between the custom inserted BamHI and NotI restriction sites. The following oligonucleotide was used: 5′-GGATCCGGTGGTGGCGGCTCTGGTGGCGGTGGCAGCCAGCTGTATGAAAATAAACCCAGAGGCCCTACATTCTCTGAGCGGCCGC-3′. The oligonucleotide duplex was inserted with standard restriction cloning. The sequence identity was verified at the UW-Madison sequencing facility and had the final annotation Clone ID-scFv-Fc-LL-NT. Fused proteins were produced and purified exactly as previously described [9]. The functional activity of neurotensin was monitored for each batch with the Path-Hunter^®^ eXpress NTSR1 CHO-K1 β-Arrestin GPCR assay (Eurofins/DiscoverX, #93-0280E2CP0L, Fremont, CA, USA), according to the manufacturer’s protocol.

### 2.2. Microvascular Endothelial Cell Immunocytochemistry

**Figure 1:**
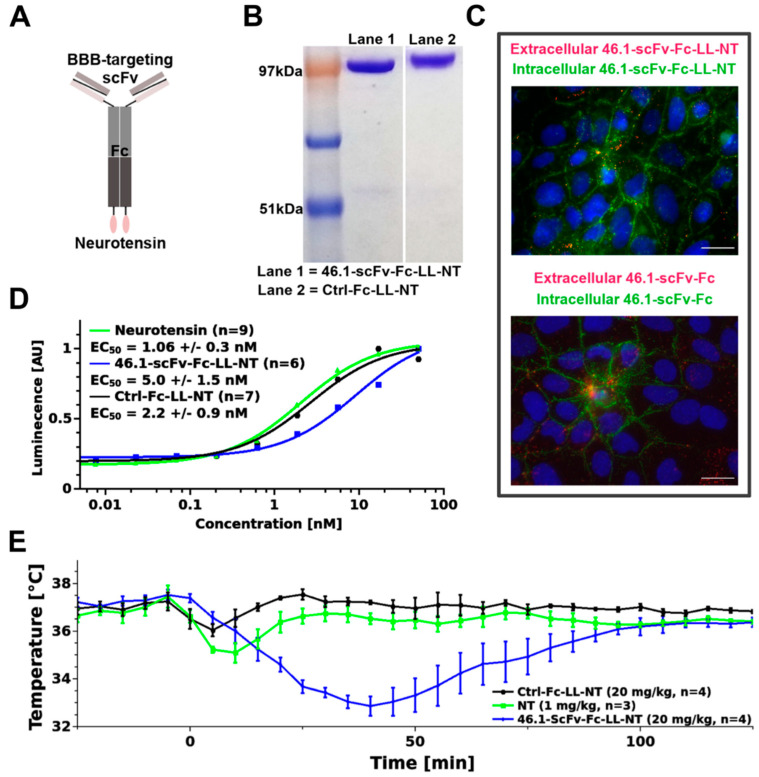
ScFv 46.1 transports neurotensin into the MnPO. (**A**) Cartoon of the antibody-NT construct. (**B**) Purified fusion constructs resolved with SDS-PAGE and Coomassie staining. (**C**) Conjugation of neurotensin to the parent antibody (lower image) has no influence on the binding- and internalization activity of 46.1-scFv-Fc-LL-NT (upper image) in iPSC-BMEC-like cells, as evident from both antibodies having a characteristic punctate appearance on the plasma membrane (pseudocolored in red) and localization to the internal cell–cell contacts (pseudocolored in green). Scale bar, 20 µm. (**D**) NT alone or antibody-NT-fusions (control and 46.1 antibody) were added in serial dilutions to NTSR1 (G-protein coupled receptor (GPCR)) expressing CHO-cells. The activation of NTSR1 was measured as a chemiluminescent light, produced by active β-galactosidase, which formed upon the recruitment of β-arrestin to the NTSR1 construct. The inset provides the fitted EC_50_ values (mean ± s.e.m.). The curves represent one biological replicate and n is listed in the inset. One-way ANOVA was used to analyze the data. The EC_50_ value for 46.1 fusion differs significantly from the EC_50_ of neurotensin with a Holm corrected *p*-value = 0.009 (Table A1). (**E**) Mice were injected at time 0 via the retro-orbital sinus with the control and 46.1 fusions or free neurotensin and the temperature recorded with an intraperitoneally-implanted probe, as described in the Materials and Methods Section. Mixed ANOVA was used to model the data (mean ± s.e.m.). The Holm–Bonferroni post hoc multiple comparisons test revealed a statistically significant difference between the 46.1-scFv-Fc-LL-NT group vs. the control (*p* = 0.001) and NT (*p* < 0.05) (Table A2).

IPSC-derived brain microvascular endothelial cells (iPSC-BMEC-like cells) were purified on Lab Tek II chamber slides (Nunc #154917, Thermo Fisher Scientific, Madison, WI, USA) (Figure 1C) or on 0.4 µm Transwell filters (Figure A2), as described previously [27,28]. Cells grown on chamber slides were washed once and the media exchanged with prewarmed PBS^++^ (PBS supplemented with Ca^2+^ and Mg^2+^). Antibody 46.1-scFv-Fc-LL-NT was added to the PBS^++^ to a final concentration of 5 µg/mL. Cells were kept at 37 °C for 45 min to allow for antibody binding and internalization. Afterward, cells were washed with ice cold PBS^++^ and media exchanged with ice cold PBS^++^ plus 10% goat serum (PBSG). Secondary anti-rabbit AlexaFluor555 (Invitrogen #A21428, Thermo Fisher Scientific, Madison, WI, USA)-conjugated antibody of a final dilution 1:1000 was added to the live cells to label the membrane bound fraction. After an additional 20 min on ice, the cells were washed three times with cold PBS^++^, fixed with ice cold 4% paraformaldehyde (PFA) for 15 min, permeabilized with 0.2% Triton X for 2 min, and washed with PBS^++^. Anti-rabbit AlexaFluor488 (Invitrogen #A11008, Thermo Fisher Scientific, Madison, WI, USA) diluted 1:1000 in PBSG was added to the chamber slides to label the internalized fraction and incubated at RT for an additional 20 min. The IPSC-BMECs were washed and mounted with ProLong Gold anti-fade reagent with DAPI (Invitrogen, P36935, Thermo Fisher Scientific, Madison, WI, USA). Images were acquired on a Zeiss Axio Imager Z2 Upright microscope (Carl Zeiss Microscopy, LLC, White Plains, NY, USA) and processed with ImageJ (Version 1.53e, Wayne Rasband and contributors, NIH, USA, http://imagej.nih.gov/ij).

The TEER value of iPSC-BMECs, grown on Transwell filters (n = 3) was measured and on the day of the experiment averaged ~1600 Ω/cm^2^. Cells were washed and media exchanged with prewarmed PBS^++^. Antibody 46.1-scFv-Fc-LL-NT was added to the basolateral compartment to a final concentration of 5 µg/mL. The IPSC-BMEC-like cells were kept at 37 °C for 45 min. After a brief washing step with ice cold PBS^++^ and PBSG, the apical and the basolateral compartment solution was exchanged with ice cold PBSG containing secondary anti-rabbit antibodies in a 1:1000 dilution, AlexaFluor647 (Invitrogen #A-21235, Thermo Fisher Scientific, Madison, WI, USA) in the apical compartment to label the apical membrane associated 46.1-scFv-Fc-LL-NT and AlexaFluor555 (Invitrogen #A21428, Thermo Fisher Scientific, Madison, WI, USA) in the basolateral compartment to label the basolateral membrane bound 46.1-scFv-Fc-LL-NT. The live, not-permeabilized cells were kept on ice for 20 min. The filter was washed quickly 3x with cold PBS^++^. Cells were fixed with ice cold 4% PFA for 15 min and permeabilized with 0.2% Triton X for 2 min, applied on both sides of the filter. Anti-rabbit AlexaFluor488 (Invitrogen #A11008, Thermo Fisher Scientific, Madison, WI, USA) in 1:1000 dilution was added to both sides of the filter to label the internalized 46.1-scFv-Fc-LL-NT and incubated at RT for an additional 20 min. Cells were washed and mounted with the ProLong Gold mounting media. Images were acquired on a Zeiss Axio Imager Z2 upright microscope and processed with ImageJ.

### 2.3. Animal Experiments

Animal studies were approved by the Institutional Animal Care and Use Committee (IACUC) at the University of Wisconsin-Madison and performed in compliance with the National Institutes of Health Guide for the Care and Use of Laboratory Animals. Mice were group housed in an AAALAC accredited vivarium on a standardized light cycle (lights on: 8 a.m.–8 p.m.) with ad libitum access to food and water.

### 2.4. Logger Implantation and Temperature Measurement

Mice C57BL6 (Harlan Laboratories, Inc., Envigo Bioproducts, Inc., Madison, WI, USA), male, ~18 g were anesthetized with 100/10 mg/kg ketamine/xylazine (Vetaket C-III (N), Akorn, Inc., #2010020, Lake Forest, IL, USA). Under aseptic conditions, an incision was made in the peritoneal cavity and a sterile temperature recording logger was inserted (DST nano-T, StarOddi, Garðabær, Iceland). The incision was sutured with a vicryl suture (Ethicon, #J422H, Bridgewater NJ, USA) and the skin glued with the tissue adhesive Vetbond (3M, #70200742529, Maplewood, MN, USA). Twenty minutes prior to recovery from anesthesia, mice received s.c. 1 mg/kg Buprenorphine SR Lab 0.5 mg/mL (ZooPharm, Laramie, WY, USA). Mice were allowed to fully recover after surgery before being transferred to their home cages. On day 6 after logger implantation, the mice were injected via the retro-orbital sinus (under brief isoflurane anesthesia, <2 min) or tail vein with 20 mg/kg control and test antibody, and 1 mg/kg neurotensin dissolved in PBS (Sigma-Aldrich, #N6383, Burlington, MA, USA). For the duration of the temperature recordings, mice were returned to their home cages. 

### 2.5. Locomotor Activity Measurement

Mice C57BL6 (Harlan Laboratories, Inc., Envigo Bioproducts, Inc, Madison, WI, USA), male, ~18 g were housed under normal conditions for 5 days. On the sixth day, each mouse was transferred to a clean plastic open field apparatus (25 cm W × 40 cm D × 20 cm H) in the recording room. Light intensity was adjusted to ~55 lux. Mice were habituated to the apparatus for 60 min prior to treatment. Animals received, via tail vein, 20 mg/kg test antibody, 1 mg/kg PD149163 (Sigma-Aldrich, #PZ0175), 1 mg/kg neurotensin, or saline. All animals also received 3 mg/kg phencyclidine (Sigma-Aldrich, #P3029) s.c. Ten minutes later, mice were returned in the same, freshly ethanol wiped apparatus and their activity was recorded for an additional 90 min. Data were collected and the distance traveled measured with ANY-maze software (Stoelting Co., Wood Dale, IL, USA). The number of animals per group is specified in the figure legends. 

### 2.6. Surgery and Cavernous Sinus Immunohistochemistry

Mice C57BL6 (Harlan Laboratories, Inc., Envigo Bioproducts, Inc, Madison, WI, USA), male, ~20 g were anesthetized with 100/10 mg/kg ketamine/xylazine and fully prepped for whole body cardiac perfusion. Test or control antibody (20 mg/kg) was injected in the retro-orbital sinus. The perfusion pump was switched on immediately and the right atrium perforated right after. To keep the tissue metabolically active, the perfusion buffer was artificial cerebrospinal fluid (119 mM NaCl, 26.2 mM NaHCO_3_, 2.5 mM KCl, 1 mM NaH_2_PO_4_, 1.3 mM MgCl_2_, 10 mM glucose, 2.5 mM CaCl_2_) kept at 37 °C for the 15 min duration of the perfusion at 1.7 mL/min. Afterward, the perfusion buffer was exchanged with ice-cold 4% PFA in PBS for an additional 5 min at rate 5 mL/min. Animals were decapitated, the skull skinned, and placed in ice-cold 12% EDTA, pH = 7. The decalcification of the skull continued for 7 days by refreshing the 12% EDTA solution every other day. The skulls were washed once with PBS, dried on a tissue paper, and snap frozen in liquid nitrogen. Consecutive coronal sections (30 µm) spanning the cavernous sinus were made on a Thermo Scientific Microm HM 525 (Thermo Fisher Scientific, Waltham, MA, USA). Sections were air dried for 1 h, permeabilized with 0.05% saponin for 30 min, and blocked with 10% goat serum in PBS for 30 min at RT. To visualize the endothelial cells and blood vessels, the sections were incubated for 2 h at RT with rat anti-mouse CD31 (Biolegend, #102501, San Diego, CA, USA) diluted 1:50 in dilution buffer (10% goat serum with 0.05% saponin in PBS). Sections were then washed 5 × with 0.05% saponin in PBS and incubated for 2 h at RT with the goat anti-mouse AlexaFluor488-conjugated secondary antibody (Invitrogen, #A-11029) 1:1000 in dilution buffer. They were washed 5 × more and incubated with the goat anti-rabbit AlexaFluor555-conjugated secondary antibody (Invitrogen, #A21428) that recognizes the rabbit Fc region of the control and test antibodies, diluted 1:1000, overnight at 4 °C. After 5 × more washing steps, sections were mounted with ProLong Gold antifade reagent with DAPI (Invitrogen, P36935) and imaged on a Zeiss Axio Imager Z2 Upright microscope or Nikon A1R HD Upright Multi-Photon (Nikon USA, Melville, NY, USA), and processed with ImageJ.

### 2.7. Statistical Analysis

Two-way repeated measures (mixed) ANOVA and Bayesian (mixed) ANOVA with treatment as the between subjects factor and time as the within subjects factor were used to model the hyperlocomotor data, which were grouped into 5 min blocks across the entire trial period. Average baseline locomotion for each individual subject was calculated across all 5 min blocks from 0 to 60 min. Hyperlocomotion data were transformed to a percent of the average baseline locomotion for each independent animal to correct for underlying individual variance in locomotor behavior. The p values by the multiple comparisons post hoc test were corrected with Holm–Bonferoni. Statistical analysis was performed with Jamovi (The Jamovi project, 2020, version 1.6, retrieved from https://www.jamovi.org, accessed on 2 March 2021). *p* values < 0.05 were considered as statistically significant. The posterior odds and Bayes factors were calculated with JASP software (JASP Team 2020, version 0.14.1, retrieved from https://www.jasp-stats.org, accessed on 3 March 2021). The posterior odds were corrected for multiple testing by fixing to 0.5 the prior probability that the null hypothesis holds across all comparisons [29]. Individual comparisons were based on the default *t*-test with a Cauchy (0, r = 1/sqrt(2)) prior. The “U” in the Bayes factor denotes that it is uncorrected.

## 3. Results

### 3.1. ScFv 46.1 Mediates the Transport of Neurotensin across the Blood Brain Barrier and Its Accumulation in the Median Preoptic Nucleus

Intracisternal administration of NT results in a decrease in the core body temperature through interactions with neurotensin receptors in the MnPO [30]; and thus, a decrease in body temperature can be used as a readout of the brain penetrating properties of RMT targeting reagents. Hence, we constructed an scFv-NT fusion protein. Neurotensin (NT) was linked via the (Gly_4_Ser)_2_ linker (LL) to the C-terminus of scFv-Fc fusions with 46.1 scFv or a control binding domain, a variable lymphocyte receptor that binds to human H antigen trisaccharide [31], to create 46.1-scFv-Fc-LL-NT and Ctrl-Fc-LL-NT fusions, respectively (Figure 1A). The fusions were produced and purified (Figure 1B), and the activity of the binding and NT moieties were confirmed. The 46.1-scFv-Fc-LL-NT fusion bound and internalized into induced pluripotent stem cell-derived BMEC-like cells (iPSC BMEC-like cells), the original cell type that was used to identify 46.1, similar to that of the parent 46.1-scFv-Fc format (Figure 1C). In particular, scFv 46.1 drives the trafficking and internalization of the parent and the NT-fused antibody in iPSC-BMEC-like cells to the intracellular cell–cell junctions as previously described (Figure 1C, green) [9]. The activity of NT in the fusion protein was measured using the PathHunter^®^ eXpress NTSR1 CHO-K1 β-Arrestin GPCR assay. Figure 1D depicts the EC_50_ values indicating a slightly diminished activity of NT when fused to the 46.1-antibody, but potency remains in the nanomolar range. Next, the Control-, scFv-46.1- fusions (20 mg/kg, ~194 nmol/kg), and NT alone (1 mg/kg, 598 nmol/kg NT) were administered via the retro-orbital sinus in C57BL6 mice. 46.1-scFv-Fc-LL-NT caused a significant transient reduction in the body temperature compared to Ctrl-Fc-LL-NT or free NT (Figure 1E) (mixed model ANOVA, F_Treatment_ (2, 8) = 17.6, *p* = 0.001), providing evidence for the scFv 46.1 mediated delivery of NT to postvascular cells in the MnPO. 

### 3.2. ScFv 46.1 Mediates the Delivery of Neurotensin to the Striatum

**Figure 2:**
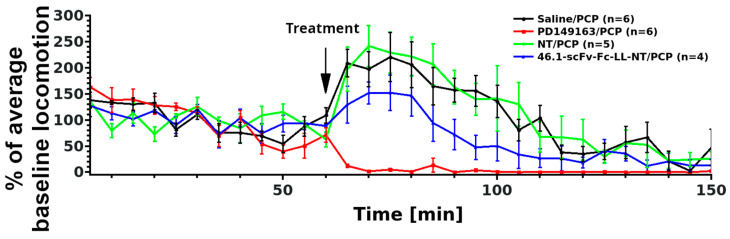
ScFv 46.1 affects NT impact on PCP-induced hyperactivity in mice. Animals were habituated in the recording setting for 1 h (0–60). The recording was stopped and saline, PD149163 (1 mg/kg), NT (1 mg/kg), or 46.1-scFv-Fc-LL-NT (20 mg/kg) were injected via the tail vein. Ten minutes later, this was followed by subcutaneous injection of PCP (3 mg/kg) to induce hyperactivity and the recording was resumed (arrow). The distance traveled is presented as a percent of the average baseline for each animal (mean ± s.e.m.). Frequentist mixed ANOVA and Bayesian mixed model ANOVA were used to analyze the data. Detailed statistical parameters can be found in Table 1 and Table 2.

NT is a well-known modulator of dopaminergic neurotransmission. Notably, spontaneous locomotor activity can be decreased by NT through interactions with dopaminergic neurons in the nucleus accumbens either by directly expressing NTSR1 or by communicating with NTSR1-responsive neurons or by receiving inhibitory signals from the ventral tegmental area [17,18]. Inspired by previous studies with BBB-permeable brain-penetrating NT analogs [32,33] that demonstrated the reversal of phencyclidine (PCP)-induced hyperlocomotor activity, and in analogy to other psychostimulants [34,35] pointing toward the activation of striatal NTSR1 in the nucleus accumbens [36] and caudate-putamen [37] as a mechanism for this effect, we explored whether scFv 46.1-mediated NT delivery would have an effect on PCP-induced changes in locomotion (Figure 2).

To avoid any interference of isoflurane anesthesia (necessary for retro-orbital sinus injection) on the effects of PCP administration, all substances were administered via tail vein injection in unanesthetized mice. The distance traveled was used as a readout of mouse locomotor activity. PCP (3 mg/kg, s.c.) was introduced 10 min after prophylactic treatment with 46.1-scFv-Fc-LL-NT or controls. The BBB permeable NT analog, PD149163, was used as a positive control, and saline and NT alone were used as the negative controls. Mixed ANOVA of the baseline-normalized, PCP-induced hyperlocomotor effects from 0–90 min identified a significant treatment effect (F_Treatment_(3, 17) = 13, *p* < 0.001). Subsequent post hoc testing could not discriminate the 46.1-scFv-Fc-LL-NT treatment group from the other groups at a significance level of α = 0.05. The associated adjusted and non-adjusted *p* values are shown in Table 1.

We additionally used Bayesian mixed ANOVA to assign the probability of differentiating between the experimental groups. Table 2 reports the posterior odds and the Bayes factors (BF_10_) in multiple comparison tests. The Bayes factors (BF_10_) calculated for the positive control PD149163 (vs. saline: BF_10_ = 2.64 × 10^20^, vs. NT: BF_10_ = 2.5 × 10^19^, vs. 46.1-scFv-Fc-LL-NT: BF_10_ = 1.36 × 10^13^) indicate extreme evidence in favor of the alternative hypothesis (i.e., PD149163 reverts the hyperlocomotion of PCP compared to any other group). The Bayes factors (BF_10_) for the experimental treatment 46.1-scFv-Fc-LL-NT compared to saline or NT indicate very strong (BF_10_ = 70.34) and extreme (BF_10_ = 147.72) evidence for accepting the alternative hypothesis, respectively (i.e., 46.1-scFv-Fc-LL-NT reverts the hyperlocomotion of PCP compared with saline or NT groups). The interpretation of the Bayes factors as very strong or extreme follows that in [38]. Thus, 46.1-scFv-Fc-LL-NT has a distinguishable effect on the PCP-induced hyperactivity that can be assigned to its own experimental group, indicating that 46.1 can mediate transport of the NT cargo into the striatum. The effect is moderate compared to the BBB permeable PD149163, which is perhaps not surprising given that the brain bioavailability of NT in the form of 46.1-scFv-Fc-LL-NT also depends on the transcytosis of the construct across the BBB.

### 3.3. Transient Hypothermia Response Depends on the Route of Delivery

**Figure 3:**
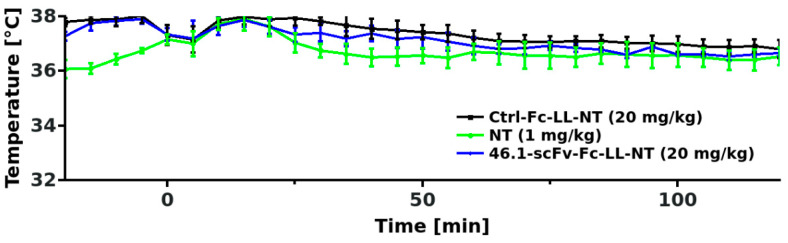
Tail vein administration abolishes the temperature response to NT. Mice (n = 4) were injected at time 0 via the tail vein with the control and 46.1 fusions or free neurotensin, and the temperature recorded with an intraperitoneally-implanted probe as described in the Materials and Methods Section. Mixed ANOVA was used to model the data (mean ± s.e.m.), F_Treatment_(2, 9) = 1.01, *p* = 0.401.

For the body temperature measurements in Figure 1, we administered the 46.1-scFv-Fc-LL-NT via retro-orbital sinus injection. We also observed that 46.1-scFv-Fc-LL-NT had a modest effect on PCP-induced hyperlocomotion when administered via the tail vein, and wondered whether the route of administration could affect the responses observed. Retro-orbital injection was not compatible with the PCP hyperlocomotion experiment as it requires brief isofluorane anesthesia prior to PCP administration. Thus, we instead explored whether or not the transient hypothermia response would be altered by tail vein administration versus retro-orbital administration. Mice received the same doses of 46.1-scFv-Fc-LL-NT and the control (20 mg/kg antibody-NT fusion or 1 mg/kg NT), as described in Figure 1 with the tail vein administration being the only difference. Unexpectedly, no discernible changes in the core body temperature were observed between the groups (Figure 3), despite the potency of PD149163 to induce hypothermia after tail vein injection (Figure A3). As detailed in the discussion below, it may be possible that there is a dilution or loss of 46.1-scFv-Fc-LL-NT as it travels from the tail vein to the heart. Alternatively, there may be a unique attribute of the retro-orbital administration paradigm with respect to the 46.1 scFv targeting system that could lead to enhanced effects in the CNS. 

### 3.4. Trans-Carotid Transport of ScFv 46.1 at the Cavernous Sinus Allows for Enhanced Accumulation of the Antibody-NT Construct in the Brain after Retro-Orbital Sinus Administration

**Figure 4:**
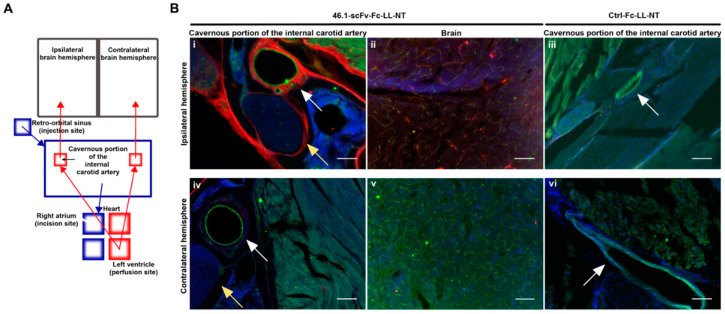
46.1-scFv-Fc-LL-NT accumulates in the cavernous sinus and brain after retro-orbital injection. (**A**) Compartmental scheme of the surgery procedure and fluid flows. Blue arrows represent the venous blood flow, red arrows the arterial flow, the black arrow represents the putative reverse transcytosis. Mice were injected with 46.1-scFv-Fc-LL-NT or Ctrl-Fc-LL-NT (20 mg/kg) in the retro-orbital sinus and immediately whole body perfused as an incision was made in the right atrium to disconnect the lung circulation. (**B**) Coronal sections of the skull at the level of the cavernous sinus were labeled for the fusion proteins with fluorescent anti-rabbit Fc AlexaFluor555 antibody (red), internal carotid artery endothelial cells (green, white arrows), and blood vessels in brain were visualized with CD31 and secondary AlexaFluor488-conjugated antibody (green). N. trigeminus (yellow arrows), nuclei (blue). Scale bar, 100 µm.

The retro-orbital sinus has direct access to the cavernous sinus. Substances introduced into the retro-orbital sinus will follow the venous blood flow to the cavernous sinus and drain through the jugular veins into the systemic venous blood, eventually reaching the heart through the superior caval vein. Likewise, substances injected into the tail vein will flow in the venous blood into the heart through the inferior caval vein. After a round of circulation through the lungs, substances, originally administered via both iv routes, will flow with the arterial blood through the carotid arteries into the brain. Unless there is a differential loss or dilution of injected material as it travels from the lateral tail vein to the heart, both intravenous routes should deliver material into the brain circulation at a comparable concentration. While not excluding the potential loss of 46.1-scFv-Fc-LL-NT after tail vein injection, we examined whether the 46.1 scFv could mediate a direct alternative uptake pathway into the brain circulation after introduction into the retro-orbital/cavernous sinuses that could possibly explain the differences observed in the body temperature responses to 46.1-scFv-Fc-LL-NT administration. In a bit more detail, before branching to enter the brain, the internal carotid arteries (left and right) span the cavernous sinus, where the outer arterial wall bathes in venous blood flowing from the retro-orbital sinus (Figure 4A). If a substance in the venous blood of the cavernous sinus can penetrate the wall of the internal carotid artery, it would directly enter the arterial flow and the brain. Figure 4A shows the experimental scheme designed to decouple antibody entry into the brain after return to the heart from a direct trans-arterial entry mechanism.

**Figure A1:**
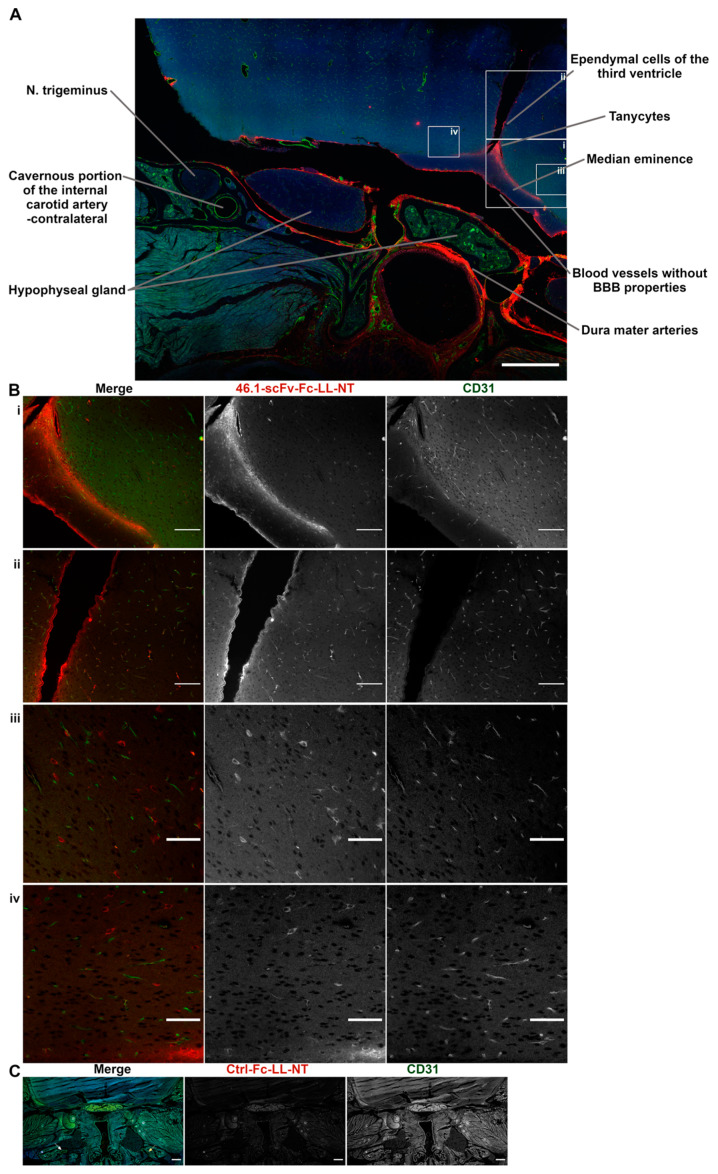
Detailed localization of 46.1-scFv-Fc-LL-NT in the cavernous sinus and brain. (**A**) Overview at low magnification of the distribution of 46.1-scFv-Fc-LL-NT (red) and CD31 (green) in the head and brain, as described in Figure 4. Scale bar, 500 µm. (**B**) Boxed areas from (**A**) (**i**) 46.1-scFv-Fc-LL-NT (red) is localized at the floor of the median eminence, tanycytes, cell bodies, and emanating projections of ME parenchymal cells. Scale bar, 100 µm. (**ii**) Hypothalamic microvasculature and ependymal cells of the third ventricle show immunoreactivity for 46.1-scFv-Fc-LL-NT. Scale bar, 100 µm. (**iii**,**iv**) Ipsilateral and contralateral hypothalamic non-vascular cells are labeled for 46.1-scFv-Fc-LL-NT. Scale bar, 50 µm. (**C**) Control antibody (red) is not detected in the head and brain. White and yellow arrows represent the cavernous portion of the internal carotid artery, contralateral, and ipsilateral, respectively. Scale bar, 500 µm.

Under deep anesthesia, mice were prepared for whole body perfusion. Ctrl-Fc-LL-NT (20 mg/kg) or 46.1-scFv-Fc-LL-NT (20 mg/kg) was injected into the retro-orbital sinus, and heart perfusion through the left ventricle began immediately after injection to assure unidirectional flow of fluid from the heart to brain, and the incision in the right atrium was made to prevent the antibody from the injection site from entering the heart. In this way, only local transport of retro-orbitally injected antibody from the venous into the arterial blood can act as a entry point to the brain circulation. To keep the tissue metabolically active, artificial cerebrospinal fluid at 37 °C was used as a perfusion buffer over the 15 min perfusion timeframe. The whole skull was processed for immunohistochemistry according to a standard procedure of bone delcalcification that preserves the brain and underlying structures of the head during sectioning (Figure 4B). In the cavernous sinus at the site of the injection (ipsilateral), multiple structures were positive for 46.1-scFv-Fc-LL-NT (Figure 4B(i), yellow arrow—N. trigeminus). The outer wall of the internal carotid artery (Figure 4B(i), white arrow) showed very strong immunoreactivity for 46.1-scFv-Fc-LL-NT. In contrast, the corresponding structures on the contralateral side (Figure 4B(iv) and Figure A1A) were negative for 46.1-scFv-Fc-LL-NT. Despite being cut off from any transport mediated by the arterial blood supply from the heart, 46.1-scFv-Fc-LL-NT reached and accumulated in brain capillaries in the ipsilateral hemisphere (Figure 4B(ii), thalamus and Figure A1B(ii), hypothalamus) but not in the contralateral hemisphere (Figure 4B(v)) other than the region, directly adjacent to the third ventricle (Figure A1B(iv)). The internal carotid artery also provides arterial blood supply for the hypophyseal gland and the median eminence (ME). We observed the accumulation of 46.1-scFv-Fc-LL-NT in both of these structures (Figure A1A), albeit only in the surrounding vessels of the hypophyseal gland. The antibody was confined within the floor of the median eminence, and multiple cell bodies and projections throughout the median eminence were also positive (Figure A1B(i)). 46.1-scFv-Fc-LL-NT also showed a very strong immunoreactivity for a subset of cells on the border of ME and the arcuate nucleus, which based on their location, were presumed to be tanycytes. Additionally, non-vascular cells in the hypothalamus, in the ipsilateral, and the contralateral hemisphere, respectively, were positive for 46.1-scFv-Fc-LL-NT (Figure A1B(iii,iv)). In comparison, Ctrl-Fc-LL-NT was not detected in the ipsilateral or contralateral cavernous sinus or in the brain (Figure 4B(iii,vi) and Figure A1C). Taken together, the data provide evidence for a first pass effect on the accumulation of 46.1-scFv-Fc-LL-NT in the CNS. After initial administration in the retro-orbital sinus, the 46.1-scFv-Fc-LL-NT is transported from the venous into the arterial blood in the cavernous sinus. Diluted in the perfusate, 46.1-scFv-Fc-LL-NT followed the natural arterial blood flow to the ME, brain capillaries, and because of its BBB-penetrating capacity, the parenchymal brain cells.

**Figure A2:**
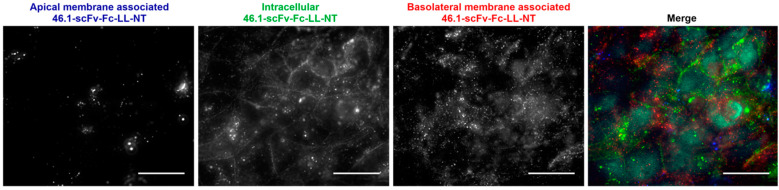
Basolateral to apical transport of 46.1-scFv-Fc-LL-NT. Confluent, barrier forming, iPSC BMEC-like cells on Transwell filters were incubated for 45 min at 37 °C with 5 µg/mL antibody 46.1-scFv-Fc-LL-NT, added to the basolateral compartment. Live cells were post-labeled on ice with secondary anti-rabbit AlexaFluor647 (blue) in the apical compartment, and AlexaFlour555 (red) in the basolateral compartment. Both compartments were washed, BMEC-like cells fixed, permeabilized, and incubated with secondary anti-rabbit AlexaFluor488 (green) to visualize the internalized antibody. All three secondary antibodies had distinctive appearances that were not spatially overlapping, indicating the lack of the paracellular diffusion of 46.1-scFv-Fc-LL-NT or any of the secondary antibodies. Of note, the intracellular fraction of 46.1-scFv-Fc-LL-NT was found at the cell–cell contacts as we have previously documented for apical to basolateral transport [9]. Scale bar, 20 µm.

**Figure A3:**
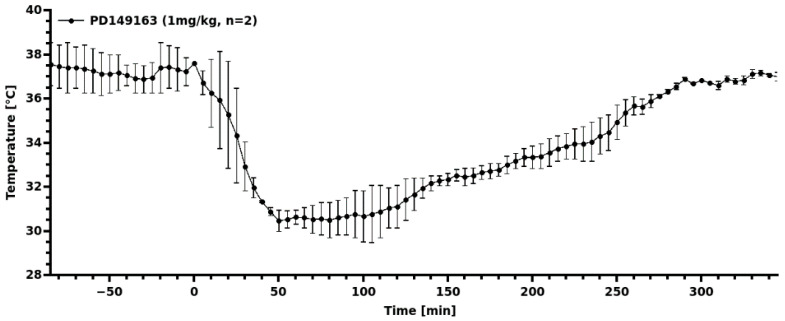
Development of hypothermia after tail vein administration of PD149163. Mice (n = 2) were injected at time 0 via the tail vein with 1 mg/kg PD149163, and the temperature recorded with an intraperitoneally-implanted probe as described in the Materials and Methods Section.

Concentration gradients between venous blood in the cavernous sinus and arterial blood in the internal carotid artery have been hypothesized to be a driving force of transport for small molecules from venous to arterial blood [39]. However, 46.1-scFv-Fc-LL-NT is a macromolecule with an approx. size of 103 kDa and gradient triggered diffusion across the endothelial wall of the carotid artery is unlikely to occur for molecules of this size. From the outer to inner, the layers of the internal carotid artery are strongly positive for 46.1-scFv-Fc-LL-NT (Figure 4B(i)), suggesting that the innermost layer of endothelial cells is the limiting transport barrier. In a previous work [9], we demonstrated the apical to basolateral receptor-mediated transcytosis of the scFv clone 46.1 across the brain endothelial cells in vivo. Here, the reverse process would be required to move 46.1-scFv-Fc-LL-NT from the basolateral side of the carotid endothelial cells to the apical side in order to enter the brain circulation. Thus, using the iPSC BMEC-like cell the in vitro Transwell system, we tested whether the 46.1-scFv-Fc-LL-NT could undergo such “reverse” transcytosis. After pulsing the 46.1-scFv-Fc-LL-NT into the basolateral compartment, it could be found bound, internalized, and trafficked to the apical side, indicating that the transport process triggered by 46.1 scFv is reversible (Figure A2). In summary, it is possible that 46.1 scFv co-opts a reverse transcytosis mechanism for rapid first pass transport from the cavernous sinus to the brain arterial blood supply.

## 4. Discussion and Conclusions

The results presented in this study are consistent with the following conclusions. First, scFv 46.1 can accumulate in the postvascular brain after intravenous administration. In particular, scFv 46.1 mediates the accumulation of NT into the MnPO and into the striatum in the form of 46.1-scFv-Fc-LL-NT fusion. Next, the route of intravenous administration affected the accumulation of 46.1-scFv-Fc-LL-NT, with the retro-orbital route leading to transient hypothermia, while 46.1-scFv-Fc-LL-NT administered via the tail vein route showed no effect. Further examination of the retro-orbital route of delivery supports a mechanism for first pass cavernous sinus to the carotid artery transport of 46.1-scFv-Fc-LL-NT, which can lead to widespread vascular distribution of fusion throughout the brain, and could be at least partially responsible for the differential effects of the administration route observed with transient hypothermia experiments.

While we have previously demonstrated the capability of the scFv 46.1 to cross the BBB and enter the brain [9], the decrease in the core body temperature evoked by 46.1-scFv-Fc-LL-NT further demonstrates that scFv 46.1 is also capable of transporting the model drug cargo, NT, across the BBB. This effect was very clear upon retro-orbital injection of the fusion protein, but we did not observe a temperature reduction when 46.1-scFv-Fc-LL-NT was administered at the same 20 mg/kg dose via the tail vein. Clearly, the bioavailability of 46.1-scFv-Fc-LL-NT in the brain capillaries of MnPO is dependent on the route of intravenous administration. While we demonstrated the local transport of the antibody from the venous and cavernous sinus to the carotid artery as one potential factor for these differences, it is also possible that loss or dilution of the antibody while it transits in venous blood from the tail vein to the heart impacts its potency once it reaches the brain circulation. Studies with contrast agents can be used to compare both routes of delivery: injected contrast media in the retro-orbital sinus flows through the superficial temporal vein, the inferior palpebral vein, and the ocular angle vein to the external jugular vein, which drains into the subclavian vein that forms the left and right superior caval veins, respectively. Injected contrast media in the lateral tail vein flows into the middle caudal vein, which merges with the left and right external iliac veins to form the inferior caval vein. Content from the superior caval veins and the inferior caval vein flows into the right atrium of the heart [40]. Thus, both administration routes merge anatomically in the right atrium of the heart for the first pass through the lungs. As scFv 46.1 also binds to the lung endothelium [9], the concentration of antibodies in the arterial blood flushing the brain may be reduced, but should be reduced to the same extent independent of the administration route. Hence, the determining factor is likely to be the concentration of antibodies reaching the right atrium, which could be a function of blood volumes, dilution differences, or antibody loss from venous blood traveling to the heart from the tail vein versus that coming from the cavernous sinus. While beyond the scope of the current study, a dose escalation study with tail vein injection could help inform as to whether the lack of response was due to antibody-NT fusion concentration differences. 

While 46.1-scFv-Fc-LL-NT did not elicit transient hypothermia after tail vein administration at 20 mg/kg, it did have an effect on PCP-induced hyperactivity. The positive control small molecule, PD149163, is brain-permeable with a Ki = 159 nM (vs. 3H-NT) for NTSR1 [41] and produced almost complete immobility in the PCP-treated mice. In contrast, 46.1-scFv-Fc-LL-NT had a more modest, but statistically distinguishable effect. While tail vein administered 46.1-scFv-Fc-LL-NT decreased PCP-induced hyperactivity in the striatum, it had no effect on transient hypothermia in the MnPO. Given the differences in these anatomically distinct brain regions and the differences in the physiologic responses, it is perhaps not unreasonable to expect differential effects. For example, a recent study suggested that the activation of the high affinity NTSR1 (Kd = 0.5 nM) on neurons is modulated by the activation of low affinity NTSR2 (Kd = 3–5 nM) on astrocytes in the median preoptic nucleus (MnPO) [19], whereas the activation of NTSR1 on neurons in the striatum is involved in the depression of PCP-induced hyperactivity. Thus, a higher concentration of NT in the MnPO would be necessary to reduce the core body temperature than in the striatum to activate NTSR1 and downstream control of locomotion, as indeed previously demonstrated [42].

Given the differences in transient hypothermia outcomes based on the route of administration and since previous studies have reported differences in the observed transduction efficiency of brain cells with the rAAV9 vector, possibly due to alternative intravenous routes of delivery [21,22], we decided to explore the potential that the 46.1 scFv could be transported in a unique way. We therefore blocked the heart circulation of retro-orbitally administered test- and control antibodies and replaced the arterial blood flow and brain circulation with a perfusion solution. With this approach, we questioned the plausibility that antibody fusions could directly access the brain circulation. Indeed, 46.1-scFv-Fc-LL-NT was readily detectable in the brain microvasculature and post-vascular brain cells. The distribution of 46.1-scFv-Fc-LL-NT in the skull and brain followed the arterial blood flow, and was largely found in the ipsilateral hemisphere consistent with a successful elimination of heart circulation of the injected antibody fusions. While such counter current transfer from the cavernous sinus to the arterial blood flow has been described for small molecules [43,44,45], likely due to purely diffusive phenomena, this would be inefficient for large protein fusions (e.g., no Ctrl-Fc-LL-NT was detected in any brain region or in the underlying cavernous sinus using this approach). Thus, after the fusion protein diffuses through the supportive layers of the internal carotid artery, we believe that the fusion protein enters the arterial blood supply through an active transport process whereby the 46.1 scFv engages its cognate RMT receptor at the carotid artery endothelial cell and transcytoses from the cavernous sinus into the arterial blood supply. Of note, in our previous study, we measured the Kd = ~150 nM of 46.1-scFv-Fc fusion and its brain concentration was ~8 nM at 1 h post retro-orbital administration (same dosage as in Figure 1). Thus, while the high concentration of antibodies in the cavernous sinus after injection favors basolateral to apical transcytosis, the concentration in the brain parenchyma is likely to be insufficient to drive significant basolateral to apical elimination of 46.1-scFv-Fc-LL-NT after brain uptake.

Once in the brain circulation, we found the 46.1-scFv-Fc-LL-NT associated with the microvasculature throughout the ipsilateral cortex. This included immunoreactivity within and outside of vessels in the hypothalamus, with postvascular cells staining positive, potentially explaining how the transient hypothermia effects are elicited within the MnPO portion of the hypothalamus. An additional interesting localization of the 46.1-scFv-Fc-LL-NT was identified in the median eminence, which is a circumventricular organ that is perfused by capillaries lacking BBB properties. Here, we observed accumulation of the fusion protein within the median eminence with high immunoreactivity at the putative tanycytes that surround the median eminence. While plausible, paracellular diffusion of 46.1-scFv-Fc-LL-NT from the median eminence as a gateway to the brain parenchyma and NTSR-neurons in the MnPO and the striatum is unlikely given that the diffusion of antibodies within the extracellular brain space is spatially limited [46].

In humans, the cavernous sinus is readily accessible via the transvenous route for routine therapeutic and diagnostic interventions [47,48]. While it would be a surgical intervention, one-time catheterization of the cavernous sinus would allow for multiple, consequent dosing, and essentially provides a new intravenous administration route compared to other invasive techniques [49]. As an alternative to the retro-orbital sinus in mice, the supratrochlear (frontal) vein of the face through the nasofrontal and superior ophthalmic vein additionally gives intravenous access to the cavernous sinus in human neonates/infants [50] and adults [51]. To our knowledge, the administration of therapeutics into the cavernous sinus has not been described thus far. However, an AAV vector with a similar administration route-dependent CNS exposure [21] is already in clinical use [52], and warrants the continued discussion of how the administration route could impact the CNS delivery, particularly with a targeting motif like scFv 46.1.

## Figures and Tables

**Table 1:** The statistical parameters for mixed ANOVA analysis of PCP-induced hyperlocomotion.

Comparison							
Treatment	Treatment	Mean Difference	SE	df	t	*p*	P_Holm_
Saline	46.1-scFv-Fc-LL-NT (20 mg/kg)	47.46	22.3	17.0	2.128	0.048	0.096
	Neurotensin (1 mg/kg)	−7.76	20.9	17.0	−0.371	0.715	0.715
	PD149163 (1 mg/kg)	105.2	19.9	17.0	5.275	<0.001	<0.001
46.1-scFv-Fc-LL-NT (20 mg/kg)	Neurotensin (1 mg/kg)	−55.22	23.2	17.0	−2.383	0.029	0.087
	PD149163 (1 mg/kg)	57.74	22.3	17.0	2.589	0.019	0.076
Neurotensin (1 mg/kg)	PD149163 (1 mg/kg)	112.96	20.9	17.0	5.400	<0.001	<0.001

**Table 2:** The statistical parameters for the Bayesian mixed ANOVA analysis of PCP-induced hyperlocomotion.

Comparison					
Treatment	Treatment	Prior Odds	Posterior Odds	BF _10, U_	Error (%)
Saline	46.1-scFv-Fc-LL-NT (20 mg/kg)	0.414	29.136	70.34	2.603 × 10^−8^
	Neurotensin (1 mg/kg)	0.414	0.074	0.178	4.210 × 10^−6^
	PD149163 (1 mg/kg)	0.414	1.093 × 10^20^	2.638 × 10^20^	3.010 × 10^−27^
46.1-scFv-Fc-LL-NT (20 mg/kg)	Neurotensin (1 mg/kg)	0.414	61.063	147.42	1.607 × 10^−9^
	PD149163 (1 mg/kg)	0.414	5.637 × 10^12^	1.361 × 10^13^	2.716 × 10^−17^
Neurotensin (1 mg/kg)	PD149163 (1 mg/kg)	0.414	1.033 × 10^19^	2.493 × 10^19^	2.053 × 10^−26^

Note. The posterior odds were corrected for multiple testing by fixing to 0.5 the prior probability that the null hypothesis holds across all comparisons [29]. Individual comparisons were based on the default *t*-test with a Cauchy (0, r = 1/sqrt(2)) prior. The “U” in the Bayes factor denotes that it is uncorrected.

## Data Availability

The data presented in this study are available in the figures and tables of the main text and Appendix A.

## Appendix A

**Table A1:** The statistical parameters for the NT activity assay.

Comparison						
Treatment	Treatment	Mean Difference	SE	df	t	P_Holm_
46.1-scFv-Fc-LL-NT (20 mg/kg)	Ctrl-Fc-LL-NT (20 mg/kg)	2.8	1.22	19.0	2.3	0.066
	Neurotensin (1 mg/kg)	3.95	1.16	19.0	3.41	0.009
Ctrl-Fc-LL-NT (20 mg/kg)	Neurotensin (1 mg/kg)	1.14	1.11	19.0	1.03	0.314

**Table A2:** The statistical parameters for the mixed ANOVA analysis of transient hypothermia.

Comparison						
Treatment	Treatment	Mean Difference	SE	df	t	P_Holm_
46.1-scFv-Fc-LL-NT (20 mg/kg)	Ctrl-Fc-LL-NT (20 mg/kg)	−1.888	0.323	8.0	−5.84	0.001
	Neurotensin (1 mg/kg)	−1.281	0.349	8.0	−3.67	0.013
Ctrl-Fc-LL-NT (20 mg/kg)	Neurotensin (1 mg/kg)	−55.22	0.349	8.0	1.74	0.12
